# Deconstructing Dissections: A Case Report and Review of Blunt Cerebrovascular Injury of the Neck

**DOI:** 10.1155/2018/6120781

**Published:** 2018-08-08

**Authors:** Brittany A. Walsh, W. Douglas Gregorie, Jessica S. Whittle

**Affiliations:** University of Tennessee College of Medicine, Chattanooga, USA

## Abstract

Blunt cerebrovascular injury (BCVI) is a term encompassing traumatic carotid and vertebral artery dissection or disruption. While the reported incidence appears to be increasing as diagnostic modalities improve, these injuries are often diagnosed only after patients have developed acute neurologic symptoms. These injuries often result in severe permanent neurologic disability or death. The gold standard for diagnosis has historically been a 4-vessel arteriogram. However, newer data are suggesting that computed tomographic angiography may be more appropriate for most patients and new criteria for its utilization have been developed. We report a case of bilateral carotid dissection in a 23-year-old woman involved in a motor vehicle collision (MVC). She initially presents with a normal neurologic exam and two hours later develops hemiparesis. She is treated with antiplatelet therapy and given intravascular catheter directed tissue plasminogen activator with carotid stent placement. Nonetheless, the patient goes on to require intubation and, ultimately, a tracheostomy and transfer to an inpatient rehabilitation setting due to continued hemiparesis. This case highlights the need for increased awareness of a potentially debilitating, life-threatening disease process. A high index of suspicion is required among emergency medicine physicians for early diagnosis and treatment of trauma patients with BCVI.

## 1. Introduction

Blunt cerebrovascular injuries (BCVI) refers to blunt traumatic carotid and vertebral artery injuries. These uncommon injuries have the potential for devastating outcomes. The following case highlights a young healthy patient who acutely develops neurologic changes secondary to bilateral BCVI after MVC. This report provides a review of the literature regarding the screening, diagnosis, and management of BCVI.

## 2. Case

A 23-year-old female was transported to the Emergency Department (ED) by ambulance after a rear-end motor vehicle collision (MVC) at highway speed. The paramedic reported she had repetitive questioning en route and complained of neck pain and left lower quadrant abdominal pain. She was placed in a cervical collar and spinal immobilization at the scene and was hemodynamically stable during transport. Based on the prehospital report, she did not meet trauma activation criteria.

On primary survey the patient was hemodynamically stable with an intact airway and normal respiratory status. She was moving all extremities equally. Initial vital signs included a blood pressure of 137/76 mmHg, heart rate of 93 beats/minute, respiratory rate of 17 breaths/minute, and temperature of 98.0°F. Secondary survey revealed a Glasgow Coma Scale (GCS) of 15 with left lower quadrant and left upper quadrant abdominal tenderness but no peritoneal signs. She was alert and oriented to person, place, and time, but she was amnestic to details of the collision. She had 5/5 strength in all extremities, and sensation was grossly intact. There were no abrasions or contusions noted to the neck, chest, or abdomen. The patient underwent computed tomography (CT) scans including brain without contrast, cervical spine without contrast, and thorax/abdomen/pelvis with contrast to assess for traumatic injuries.

**Figure 1:**
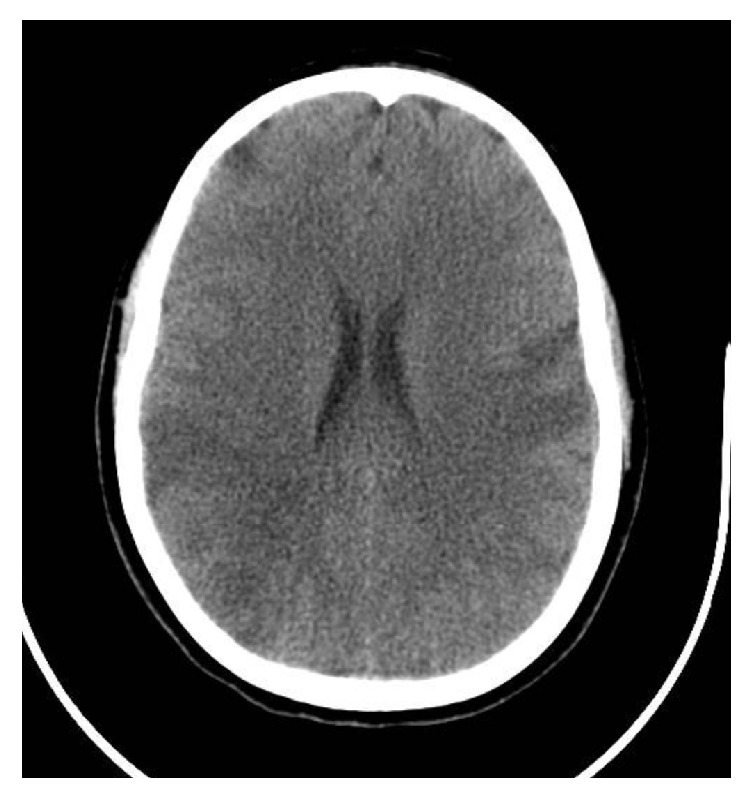
Image from the patient's CT brain without contrast demonstrating multiple acute infarcts, including right middle cerebral artery (MCA) territory.

CT scans of the brain, c-spine, and thorax/abdomen/pelvis were unremarkable with the exception of a grade III splenic laceration. Her cervical collar was removed and her c-spine clinically cleared at the bedside. Of note, she specifically denied midline tenderness to palpation and was able to move her neck in all directions without pain. She did endorse tenderness to the paraspinal muscles of the cervical spine and bilateral trapezius muscles after her collar was removed. She continued to experience repetitive questioning at that time, raising suspicion for a traumatic brain injury. The trauma service was consulted for admission and further management of her injuries. Approximately two hours after arrival, while still undergoing evaluation by the trauma team, her family noted to the ED nurse that the patient was no longer moving her right upper and right lower extremities. No facial droop was noted. CTA of the head and neck showed a right proximal internal carotid artery (ICA) occlusion and a near occlusive thrombus of the left ICA. Heparin therapy was initiated. Her GCS was notably decreasing, resulting in subsequent intubation for airway protection. CTA was followed with a confirmatory angiogram that showed an occlusion of the cervical segment of the right internal carotid artery secondary to underlying dissection and a dissection in the distal cervical segment of left internal carotid. Both middle cerebral artery (MCA) territories showed multiple areas of bilateral branch occlusions. The patient was given a loading dose of abciximab and 4 mg of tissue plasminogen activator (TPA) through the intravascular catheter prior to intervention. A stent was deployed in the left carotid artery where a large, wall-adherent thrombus was noted. CT brain without contrast obtained the following morning showed bilateral MCA infarcts (Figure 1). The patient's mental status improved slowly, but she ultimately required a tracheostomy and feeding conduit. She was transferred to an inpatient rehabilitation facility for further recovery. At the time of hospital discharge, she was able to answer questions with nods but continued to experience right sided hemiparesis.

## 3. Discussion

While the most common mechanism for sustaining BCVI is MVC, incidence rates of BCVI are estimated at less than 1% of all inpatients admitted for care of injuries sustained in motor vehicle collision (MVC) [1–7]. Of patients with blunt carotid injury, up to 30% may be bilateral [1]. Shearing of the relatively immobile vessel from acceleration/deceleration forces and hyperflexion or hyperextension of the neck cause intimal injury, and these tears can become a nidus for thrombus development [4]. Other documented mechanisms include motorcycle crashes, assault, hanging/strangulation, chiropractic manipulations of the neck, facial or skull fractures, and bungee jumping. Populations at increased risk for nontraumatic cerebrovascular injury include patients with collagen vascular diseases and women in the postpartum period.

Presentation of patients with BCVI is widely variable, ranging from nonspecific symptoms such as headache or neck pain to severe focal paralysis resulting from ischemic stroke. Compared to vertebral artery injury, carotid artery injury has a higher rate of stroke [5]. Other symptoms may include pulsatile tinnitus, transient blindness (amaurosis fugax), and partial Horner's syndrome. The partial Horner's syndrome consisting of miosis and ptosis is due to stretching of the nearby sympathetic plexus by the same force that injures the carotid. Because of the diverse constellation of symptoms, a general principle is to suspect a BCVI based on injury patterns or if the neurologic exam does not correlate with imaging findings. In one study, 43% of patients with blunt carotid injury were diagnosed with focal neurologic deficits and 34% by physical exam findings incongruent with plain head CT results [1].

The diagnostic picture of BCVI is often complicated due to the variability of signs and symptoms indicating injury. Further complication results from that variable timeframe of presentation. Time to definitive diagnosis of BCVI with angiogram is reported as an average of 53 hours from initial injury, with a range of 2-672 hours [1]. By using screening criteria in a follow-up study, time to diagnosis with angiogram was decreased to a mean of 29.8 hours [5]. These data highlight the value of the utilization of screening criteria and should be considered by all ED practitioners. Given the potentially large time-gap between injury and symptom development, BCVI should always be included in the differential diagnosis if a patient presents with new neurologic symptoms in the days following blunt trauma.

The gold standard for diagnosing BCVI (including dissections, occlusions, and pseudoaneurysm) has been accepted to be four-vessel cerebral angiogram [3, 4, 6, 8]. Like all invasive procedures, angiograms have some risks including bleeding, vessel injury, and stroke. Additionally, aggressive screening with angiography is not feasible at a majority of facilities due to staffing, cost, and labor requirements.

As an alternate testing modality, computed tomographic angiography (CTA) is time-efficient, especially for a trauma patient already destined for the CT scanner. The poor sensitivity of early generation CT scanners limited the ability of using CTA as a screening modality with sensitivities ranging from 47-52% [5, 9, 10]. Subsequent generation scanners have demonstrated improved sensitivity to 68-100% [7, 9–12]. Present recommendations indicate that an angiogram may still be warranted if there is a high suspicion based on neurologic exam findings and a negative CTA but, in the study by Berne et al [12], there were no patients with a negative CTA that were later diagnosed with BCVI. Positive CTA often requires follow-up with angiography as the positive predictive value of CTA can range from 36 to 55% [7, 10].

Other diagnostic modalities that have been considered include magnetic resonance angiography (MRA) and duplex ultrasonography. Magnetic resonance angiography has a considerably higher time-expenditure and also a lower sensitivity (47%) than angiography [5]. Furthermore, MRA is not available in many EDs, and patients with indwelling metal devices or necessary external medical equipment (i.e., orthopedic traction devices) are not candidates. Duplex ultrasonography is unable to evaluate the intracranial vessels for injury. Therefore, both the Western Trauma Association and Eastern Association for the Surgery of Trauma recommend against using duplex ultrasound as a sole screening method for BCVI [8, 9].

Because of the time-sensitive nature of the disease process and the limitations of diagnostic modalities, there have been attempts to develop clinical criteria to identify cases at high risk for BCVI that warrant screening imaging. The development of neurologic sequelae is indicative of a poor prognosis, and early initiation of anticoagulation reduces the risk of stroke.

Studies from Memphis, TN suggest that the incidence of BCVI is higher than historically reported [1, 5]. They showed that an alarming number of these injuries were occult by utilizing aggressive screening techniques with angiography to identify BCVI prior to onset of symptoms. Researchers outlined injury profiles that correlated with BCVI, noted in Table 1. They screened 216 patients with 4-vessel angiography and diagnosed 24 patients with carotid injuries and 43 patients with vertebral artery injuries.

Completed shortly after the Memphis study, another single-center study from Denver, CO, published the “Denver Criteria.” The Denver screening criteria (Table 2) are based on signs and symptoms of injury and risk factors of concordant injuries or injury patterns. High risk mechanisms that should prompt thoughtful assessment and further screening include hyperextension, rotation, or hyperflexion of the neck and direct trauma to the neck [3]. They performed angiography in 249 patients, diagnosing 85 patients with BCVI. Forty patients had signs or symptoms of potential injury at time of angiography, and 28 were diagnosed with BCVI. Of the patients that were asymptomatic that met screening criteria, 27% had a BCVI. Vertebral artery injuries correlate most significantly with the cervical spine fracture patterns outlined in Table 2 because of their anatomic proximity. In patients with a cervical spine injury, 39% had a vertebral artery injury [3]. Trauma association management guidelines utilize the Denver criteria as a basis for screening patients for BCVI [8, 9].

Patient management goals focus on minimizing secondary neurologic deficits. Anticoagulation with heparin or other anticoagulants is currently the mainstay of therapy. The intention of heparin therapy is to diminish propagation of the clot(s) present on damaged intima, prevent additional clot formation, and reduce embolic complications. Ideally, anticoagulation is started before the onset of neurologic symptoms to minimize morbidity and mortality. Heparin therapy decreases morbidity and mortality by reducing stroke rate [1, 5, 6]. It may improve neurologic outcomes in patients that have developed symptoms [1]. In trauma patients, systemic anticoagulation may increase risk for bleeding, depending on other injuries sustained. Anticoagulation is preferable for lesions not amenable to surgery, such as intracranial lesions or those near the carotid bifurcation.

Current recommendations include initiating a heparin infusion without a bolus at a dose of 10 u/kg/hr with target PTT of 40-50 seconds due to high risk of hemorrhagic complications [9, 13]. Antiplatelet therapy, such as aspirin or clopidogrel, may be an alternative option, especially for patients with a relative contraindication to anticoagulation [5, 6, 8, 9]. Further large-scale, randomized clinical trial data is needed comparing heparin and antiplatelet agents directly. Duration of anticoagulation or antiplatelet therapy is at the discretion of the treating team and may be lifelong if a lesion persists. Surgical approaches are utilized less often, mostly for high-grade lesions (i.e., transection with extravasation). Techniques include ligation, resection of damaged areas of vessel with replacement grafts, or bypassing the affected region [1].

Endovascular techniques are being employed more often to manage vascular trauma, including stents for carotid injuries [7, 14]. The goal of a stent is to increase laminar flow through a vessel and eliminate a false lumen in the case of a pseudoaneurysm [15]. This is especially optimal in lesions difficult to access with open techniques. Endovascular treatment requires specialty care and may necessitate transfer if not available.

Patients with BCVI may present with symptoms acutely up to several weeks after the initial insult. This timeframe, combined with variable symptomatology in the presentation of BCVI, creates an obvious challenge for the emergency medicine physician. Careful attention to the risks of BCVI based on mechanism of injury and associated injury patterns is required. A combination of elevated clinical suspicion and earlier diagnosis with screening CTA may lead to earlier interventions and improved outcomes. In patients who have developed symptoms, systemic anticoagulation, typically with heparin, has been shown to improve outcomes. Endovascular procedures for blunt carotid injury management are increasingly utilized to improve outcome and treat symptomatic patients.

Currently adopted guidelines have sought to capture as many of this type of injury as possible while maintaining balance in regards to resource utilization. The patient presented in this report is atypical in that repetitive questioning was the only symptom of any neurologic process at the time of her arrival to the Emergency Department. However, her story highlights the need for careful re-evaluation of patients, elevated clinical suspicion for BCVI, and the challenges associated with identifying occult injury. This case highlights the need for additional research to refine guidelines for optimal diagnosis and treatment.

## Figures and Tables

**Table 1:** Memphis criteria screening for BCVI.

Cervical spine fracture
Horner's syndrome
LeFort II or III facial fractures
Neck soft tissue injury (i.e., hanging, seat belt, and visible hematoma)
Neurologic exam not explained by brain imaging
Skull base fractures (involving carotid canal)

**Table 2:** Screening criteria for BCVI from Denver.

Signs/symptoms of injury	Risk factors for BCVI
	*High energy mechanism with:*
Arterial hemorrhage (i.e., from neck, nose, and mouth)	Basilar skull fracture with carotid canal involvement
Cervical bruit Expanding cervical hematoma Focal neurological deficit Ischemic stroke on secondary CT scan	Cervical spine fracture patterns (i) Subluxation (ii) Fractures extending into transverse foramen (iii) Fractures of C1-C3
	Diffuse Axonal Injury GCS ≤ 6
	LeFort II-III fracture
	Near-hanging with anoxic brain injury

## References

[1] Fabian T. C., Patton J. H., Croce M. A., Minard G., Kudsk K. A., Pritchard F. E. (1996). Blunt carotid injury: importance of early diagnosis and anticoagulant therapy. *Annals of Surgery*.

[2] Baker W. E., Wassermann J. (2004). Unsuspected vascular trauma: Blunt arterial injuries. *Emergency Medicine Clinics of North America*.

[3] Biffl W. L., Moore E. E., Offner P. J. (1999). Optimizing screening for blunt cerebrovascular injuries. *The American Journal of Surgery*.

[4] Heuer G. G., LeRoux P. D., Hurst R. W. (2011). Blunt Cerebrovascular Injury. *Youmans Neurological Surgery*.

[5] Miller P. R., Fabian T. C., Croce M. A. (2002). Prospective screening for blunt cerebrovascular injuries: analysis of diagnostic modalities and outcomes. *Annals of Surgery*.

[6] Miller P. R., Fabian T. C., Bee T. K. (2001). Blunt cerebrovascular injuries: diagnosis and treatment. *Journal of Trauma, Injury, Infection, and Critical Care*.

[7] Shahan C. P., Magnotti L. J., Stickley S. M. (2016). A safe and effective management strategy for blunt cerebrovascular injury: Avoiding unnecessary anticoagulation and eliminating stroke. *Journal of Trauma and Acute Care Surgery*.

[8] Bromberg W. J., Collier B. C., Diebel L. N. (2010). Blunt cerebrovascular injury practice management guidelines: The eastern association for the surgery of trauma. *Journal of Trauma - Injury Infection and Critical Care*.

[9] *Screening for and treatment of blunt cerebrovascular injuries algorithm*.

[10] Paulus E. M., Fabian T. C., Savage S. A. (2014). Blunt cerebrovascular injury screening with 64-channel multidetector computed tomography: more slices finally cut it. *Journal of Trauma and Acute Care Surgery*.

[11] Biffl W. L., Egglin T., Benedetto B. (2006). Sixteen-slice computed tomographic angiography is a reliable noninvasive screening test for clinically significant blunt cerebrovascular injuries. *Journal of Trauma - Injury Infection and Critical Care*.

[12] Berne J. D., Reuland K. S., Villarreal D. H., McGovern T. M., Rowe S. A., Norwood S. H. (2006). Sixteen-slice multi-detector computed tomographic angiography improves the accuracy of screening for blunt cerebrovascular injury. *Journal of Trauma - Injury Infection and Critical Care*.

[13] Biffl W. L., Ray C. E., Moore E. E. (2002). Treatment-related outcomes from blunt cerebrovascular injuries: importance of routine follow-up arteriography. *Annals of Surgery*.

[14] Avery L. E., Stahlfeld K. R., Corcos A. C. (2012). Evolving role of endovascular techniques for traumatic vascular injury. *Journal of Trauma and Acute Care Surgery*.

[15] Cothren C. C., Moore E. E., Ray C. E. (2005). Carotid artery stents for blunt cerebrovascular injury: risks exceed benefits. *JAMA Surgery*.

